# Measuring health literacy to inform actions to address health inequities: a cluster analysis approach based on the Australian national health literacy survey

**DOI:** 10.1093/pubmed/fdae165

**Published:** 2024-08-04

**Authors:** Christina Cheng, Shandell Elmer, Roy Batterham, Melanie Hawkins, Richard H Osborne

**Affiliations:** Centre for Global Health and Equity, School of Health Sciences, Swinburne University of Technology, Hawthorn 3122, Victoria, Australia; School of Nursing College of Health and Medicine, College of Health and Medicine, University of Tasmania, Launceston 7250, Tasmania, Australia; Global Health Program, Faculty of Public Health, Thammasat University, Bangkok 10200, Thailand; Centre for Global Health and Equity, School of Health Sciences, Swinburne University of Technology, Hawthorn 3122, Victoria, Australia; Centre for Global Health and Equity, School of Health Sciences, Swinburne University of Technology, Hawthorn 3122, Victoria, Australia

**Keywords:** cluster analysis, health equity, health literacy, measurement, methods

## Abstract

**Background:**

Measuring health literacy can inform interventions to address health inequities. This study used cluster analysis to examine health literacy data to determine if it can provide more insightful information than standard descriptive analysis to better inform intervention development.

**Methods:**

Using data from the Australian National Health Survey (2018), this study compared descriptive analysis and cluster analysis results of two states—New South Wales (NSW) and Victoria—generated from the Health Literacy Questionnaire (HLQ). Based on the nine scale scores of the HLQ, a hierarchical cluster analysis using Ward’s method for linkage was undertaken.

**Results:**

The number of NSW and Victoria respondents was 1018 and 923, respectively. The nine HLQ scale full sample mean scores from both states were similar. However, the cluster analyses identified 11 clusters for NSW and 12 clusters for Victoria. While six clusters from each state presented similar health literacy patterns, five and six clusters from NSW and Victoria, respectively, displayed unique health literacy patterns.

**Conclusions:**

The results demonstrate that descriptive analysis only provides an overview and may lead to one-size-fits-all interventions. The varying health literacy patterns among subgroups resulting from the cluster analysis pave the way to inform tailored actions to improve health equity.

## Introduction

The World Health Organization (WHO) Shanghai Declaration in 2016 recognized that health literacy is a critical determinant of health. It further called for national and local strategies to enhance health literacy in all populations.^1^ As a public health practice, health literacy is intrinsically linked to health equity^2^^,^^3^ and is an important step to inform interventions to address health inequities.^4^^,^^5^ However, little attention is paid to how the scoring of health literacy tools or analytical methods can be used to inform intervention development.

Health literacy, a multi-dimensional concept, recognizes that people have different capacities and experiences in approaching and using health information and services.^3^^,^^4^ It refers to ‘people’s knowledge, confidence and comfort—which accumulate through daily activities and social interactions and across generations— to access, understand, appraise, remember and use information about health and health care, for the health and wellbeing of themselves and those around them’.^3^^,^^6^^,^^7^ Although many tools have been developed to understand health literacy of populations, the Health Literacy Questionnaire (HLQ) is a well-developed one that has been widely used to inform intervention development using a strengths-based approach.^6^ In 2018, the Australian Bureau of Statistics (ABS) applied the HLQ within the country’s National Health Survey.

The HLQ was developed using a co-design approach in consultation with community members, professionals and policy makers.^8^ Robust evidence about the construct validity and reliability of inferences drawn from the HLQ data has been demonstrated in numerous studies^8^^,^^9^ across various languages.^10–12^ The 44-item questionnaire has nine scales representing nine health literacy dimensions (see supplementary materials Table S1 for dimension descriptions): (i) Feeling understood and supported by healthcare providers. (ii) Having sufficient information to manage my health. (iii) Actively managing my health. (iv) Social support for health. (v) Appraisal of health information. (vi) Ability to actively engage with healthcare providers. (vii) Navigating the healthcare system. (viii) Ability to find good health information. (ix) Understand health information well enough to know what to do.^8^

While most health literacy tools present their results as an overall average score, followed by an arbitrary classification of respondents into high or low,^13^^,^^14^ the results of the HLQ are presented as nine scale scores. The HLQ does not classify people as having high or low health literacy because this may lead to stigmatization. The HLQ is built on the principle that people are different and the scoring allows for a strengths-based approach to intervention development by generating data about people’s diverse and varying health literacy strengths, challenges and preferences across nine dimensions of health literacy. As such, people’s strengths and preferences can be considered when tailoring responsive actions to meet people’s health literacy challenges.

The HLQ scales have four to six items. The response option for Scales 1 to 5 is a 4-point ordinal scale of ‘strongly disagree’ to ‘strongly agree’ (score range: 1 to 4), whereas Scales 6 to 9 have a 5-point ordinal scale of ‘cannot do or always difficult’ to ‘always easy’ (score range: 1 to 5). Each scale score is calculated by averaging the item scores with equal weighting.^8^

A total of 5790 participants completed the population-based Australian Health Literacy Survey. The mean scores for Scales 1 to 5 ranged from 2.92 (SD 0.45) (Scale 5) to 3.19 (SD 0.49) (Scale 1) while mean scores for Scales 6 to 9 ranged from 4.02 (SD 0.62) (Scale 7) to 4.29 (SD 0.56) (Scale 9).^15^ The results led the ABS to conclude that most Australians reported ‘feeling positive’ about their health literacy.^16^ However, people are different and heterogeneity exists among all populations.^17^^,^^18^ Standard descriptive analysis using full sample mean scores may mask the heterogeneity of scores across the different health literacy dimensions within a population, leading to one-size-fits-all interventions and potentially exacerbating health inequities.

To identify subgroups based on their responses to a set of variables, cluster analysis is a statistical method commonly used in marketing research^19^ and now increasingly used in public health research since the introduction of the HLQ.^20–24^ The purpose of cluster analysis is to ‘maximize the homogeneity of objects within the clusters while also maximizing the heterogeneity between the clusters’.^25^ It is a technique that is independent of scoring thresholds, which can be arbitrarily imposed by questionnaire developers.^26^ To gain a deeper understanding of the health literacy across different sub-populations, this study used the data from the 2018 ABS Health Literacy Survey and applied cluster analysis to two population groups within the dataset. The aim was to determine if cluster analysis can provide more insightful information about people’s health literacy than standard descriptive analysis, and therefore better inform health literacy actions that are tailored and fit-for-purpose to address health inequities for broader public health application.

## Methods

### Study design and participants

The ABS Health Literacy Survey was conducted from January to August 2018. Respondents were recruited from the National Health Survey, who were aged 18 and consented to participating in further surveys. The sample from the National Health Survey included people from urban and rural areas in Australia, but very remote areas and discrete Aboriginal and Torres Strait Islander communities were not included. The Health Literacy Survey was conducted by interviewers using Computer Assisted Telephone Interviewing. The health literacy data can be combined with demographic, socioeconomic characteristics, risk factors and health conditions from the National Health Survey collected by personal interviews in sampled dwellings. A total of 7224 respondents from the National Health Survey agreed to be contacted for further activities and 5790 respondents took part in the Health Literacy Survey (response rate: 80.1%). The sample represented 35.3% of the full National Health Survey. Full details of the study design such as sampling weight and handling of missing values can be found on or requested from the ABS website.^15^

This study used data from the two most populous Australian states—New South Wales (NSW) and Victoria, with a population of 8.1 million and 6.5 million, respectively, in the 2018 census.^27^ The two states were selected for comparison because a typical person from NSW and Victoria share many similar demographic characteristics compared with other Australian states and territories (see supplementary materials Table S2).

### Statistical analysis

The statistical analysis was conducted using SPSS version 27.^28^ Descriptive statistics were used for participant characteristics and the HLQ scale scores. For the cluster analysis, there are different cluster analysis techniques, and which method to use depends on the type of variables, objective of the analysis, and the experience of the researchers.^29^^,^^30^ This study used hierarchical cluster analysis, based on the nine scale scores, which were standardized due to the different score ranges. Unlike nonhierarchical clustering procedures, such as the K-means algorithms, in which researchers have to specify the number of clusters, the hierarchical procedure allows for the exploration of cluster solutions to identify the optimal number of clusters that best represents the observations.^25^ The hierarchical procedure of this study used the agglomerative method. This method begins with each observation as a cluster and combines the most similar clusters, one at a time, until all observations become one single cluster.^25^ The clustering algorithm used was Ward’s method for linkage. This method approaches the clustering as an analysis of variance and combines clusters that have minimum within-cluster variance.^25^ For the calculation of dissimilarity information, the squared Euclidean distance, the default measure in SPSS, was used.

While the cluster analysis used the nine scale scores as the basis to identify subgroups, demographic characteristics, and variables of interest, such as health data, for each cluster were included in this analysis. These variables provide context for each cluster and help determine the optimal number of clusters. This approach to cluster analysis has been used in the Optimizing Health Literacy and Access (Ophelia) process, which uses the HLQ to identify health literacy profiles to develop vignettes, used for co-design of health literacy actions to address health inequities. The Ophelia process has been implemented in many countries and various settings, such as the WHO National Health Literacy Development Projects.^6^ Responses from the field indicate that the resulting vignettes are well-recognized by stakeholders and create engaging discussion to co-design actions to address the health literacy challenges presented in the vignettes.^21^^,^^31^ Demographics included for this analysis were age, sex, country of birth, education, employment status, income, Index of Relative Socio-Economic Disadvantage (a socio-economic index that summarizes the economic and social conditions of people within an area, as used by the ABS), area of residence (metropolitan or regional/remote areas), living alone, presence of disability, chronic health condition (including arthritis, back pain, cancer, cardiovascular disease, diabetes, respiratory disease, anxiety and depression), living with two or more chronic conditions, overweight or obese based on BMI and psychological distress (Kessler 10^32^).

To determine the optimal number of clusters, 3 to 16 cluster solutions were generated. Each cluster split was examined to determine if they were different; that is, if the two new clusters or one of the new clusters presented health literacy patterns that were different from the parent cluster. A key consideration is to determine if any single cluster is sufficiently homogenous to be considered as representing a group with relatively homogenous health literacy strengths and challenges such that meaningful action can be taken to support this group. For example, if a cluster has high remaining variance on the scale of social support, i.e., this cluster includes people with both very high and very low scores on social support, a decision needs to be made by considering if meaningful action can be taken to accommodate the people in this cluster or allowing the cluster split to make the results substantively meaningful.

There are no standard procedures to validate the selected optimal cluster solution^25^ and most recommended approaches in the literature are not applicable because the current method involves not only the cluster analysis variables, but also related demographic and health data. When two clusters presented similar patterns but represented different demographic groups, these clusters were retained if different health literacy actions were expected. For example, a cluster with a high proportion of people living in rural areas is likely to require different interventions to address challenges in accessing healthcare, compared with another cluster with people mainly living in metropolitan areas. Finally, unlike standard cluster analysis where small clusters are considered outliers and usually ignored,^25^ consideration was given to determine if these clusters might represent marginalized people. These small clusters could be representing groups experiencing social and economic exclusion due to factors such as age, gender, race, class, socioeconomic status or stigmatized identities.^33^ Marginalization has been linked to poorer health outcomes^34^ and marginalized people tend to be under-represented in most public health research.^35^ Given the possible inclusion of outliers, using method like cophenetic correlation coefficient for cluster solution validation may not be appropriate because this method is sensitive to outliers.^36^ To validate the suitability of including small clusters, discussion with frontline health professionals was undertaken to check if they recognized the health literacy patterns and related groups of their clients represented in these small clusters. If health professionals confirmed the health literacy patterns in these clusters were familiar, these clusters were kept to maximize inclusion, given that the purpose of the analysis was to improve health equity. Finally, the clusters were given a short descriptive name to improve communication to others. The final clustering solution was reviewed and discussed among the authors.

## Results

### Demographics and HLQ mean scores

A total of 1018 and 923 participants in NSW and Victoria respectively completed the Health Literacy Survey. The mean age of NSW participants was a little higher (Mean (M) = 53.4, standard deviation (SD) = 17.4) than Victoria (M = 51.0, SD = 17.6). The proportion of male and female was similar. Victoria had a higher proportion (40.0%) of people with university or above education than NSW (32.8%) while people having the lowest personal weekly income were also similar. The proportion of people living with two or more chronic conditions was similar, as well as reported psychological distress. Details of the demographic characteristics are presented in Table 1.

**Table 1:** Participants Demographics and Health Literacy Questionnaire (HLQ) Scores for Respondents from New South Wales (NSW) and Victoria

		NSW (N = 1018)	Victoria (N = 923)
**Characteristics**		**N (%)**	**N (%) **
Age (Mean, Standard deviation)		53.4 (17.4) Range: 18–92	51.0 (17.6) Range: 18–94
Sex			
	Male	471 (46.3)	432 (46.8)
	Female	547 (53.7)	491 (53.2)
Australian born		737 (72.4)	651 (70.5)
Spoke English at home		932 (91.6)	838 (90.8)
Non-indigenous status		997 (97.9)	917 (99.3)
Education			
	Did not complete secondary school	248 (24.4)	177 (19.2)
	Completed secondary school	112 (11.0)	99 (10.7)
	Diploma or Certificate	324 (31.8)	278 (30.1)
	University of above	334 (32.8)	369 (40.0)
Employed (full-time or part-time)		592 (58.2)	577 (62.5)
^a^Personal weekly income			
	First and second decile	103 (10.1)	107 (11.6)
	Third and fourth decile	237 (23.3)	199 (21.6)
	Fifth and sixth decile	221 (21.7)	205 (22.2)
	Seventh and eighth decile	201 (19.7)	204 (22.1)
	Ninth and tenth decile	223 (21.9)	182 (19.7)
Remoteness of residence			
	Major cities	787 (77.3)	684 (74.1)
	Inner regional	196 (19.3)	208 (22.5)
	Outer regional	35 (3.4)	31 (3.4)
^b^Index of Relative Socio-Economic Disadvantage (IRSD) (By State)			
	First and second decile	147 (14.4)	144 (15.6)
	Third and fourth decile	189 (18.6)	180 (19.5)
	Fifth and sixth decile	199 (19.5)	205 (22.2)
	Seventh and eighth decile	213 (20.9)	182 (19.7)
	Ninth and tenth decile	270 (26.5)	212 (23.0)
Number of household members			
	One	273 (26.8)	250 (27.1)
	Two	370 (36.3)	321 (34.8)
	Three to four	294 (28.9)	274 (29.6)
	Five or more	81 (8.0)	78 (8.5)
Presence of any type of disability		289 (28.4)	253 (27.4)
Living with a chronic condition			
	Arthritis	402 (39.5)	321 (34.8)
	Back pain	138 (13.6)	145 (15.7)
	Cancer	195 (19.2)	128 (13.9)
	Diabetes	156 (15.3)	110 (11.9)
	Heart disease	454 (44.6)	375 (40.6)
	Respiratory disease	403 (39.6)	417 (45.2)
	Anxiety	187 (18.4)	177 (19.2)
	Depression	184 (18.1)	158 (17.1)
	Other	888 (87.2)	797 (86.3)
Living with one chronic condition		168 (16.5)	155 (16.8)
Living with two chronic conditions		219 (21.5)	215 (23.3)
Living with three or more chronic conditions		577 (56.7)	508 (55.0)
Self-reported body mass index (BMI) (Mean, Standard deviation)		27.4 (5.6) Range: 15– 68	27.2 (5.8) Range: 17– 56
Self-reported BMI (categories)			
	Underweight	13 (1.3)	17 (1.8)
	Normal	321 (31.5)	300 (32.5)
	Overweight	341 (33.5)	295 (32.0)
	Obese	235 (23.1)	206 (22.3)
Kessler 10 score (Mean, Standard dDeviation)		15.2 (5.7) Range: 10– 47	15.6 (5.9) Range: 10–46
^c^Kessler 10 score (categorized)			
	Low distress	669 (65.7)	589 (63.8)
	Moderate distress	223 (21.9)	214 (23.2)
	High to very high distress	125 (12.2)	120 (13.0)
**HLQ Scales**		**Score (Mean, Standard deviation** **[95% Confidence interval)**	
		Score range: 1 (lowest)–4 (highest)	
	1. Feeling understood and supported by healthcare providers	3.21 (0.47) [3.18–3.24]	3.23 (0.50) [3.20–3.27]
	2. Having sufficient information to manage my health	3.16 (0.41) [3.13–3.18]	3.19 (0.41) [3.16– 3.21]
	3. Actively managing my health	3.10 (0.45) [3.07–3.13]	3.13 (0.45) [3.10–3.16]
	4. Social support for health	3.16 (0.45) [3.13–3.18]	3.20 (0.47) [3.16–3.23]
	5. Appraisal of health information	2.92 (0.43) [2.89– 2.95]	2.94 (0.47) [2.91–2.97]
		Score range: 1 (lowest)–5 (highest)	
	6. Ability to actively engage with healthcare providers	4.19 (0.64) [4.15– 4.23]	4.23 (0.59) [4.19– 4.27]
	7. Navigating the healthcare system	4.02 (0.62) [3.98– 4.06]	4.06 (0.58) [4.02–4.10]
	8. Ability to find good health information	4.09 (0.59) [4.05– 4.12]	4.12 (0.57) [4.09–4.16]
	9. Understand health information well enough to know what to do	4.27 (0.56) [4.24–4.31]	4.30 (0.56) [4.26–4.33]

a Based on personal weekly income Deciles 1–4, income deciles are ten populations groups classified from 1–10 in ascending order with the first decile represents the group with the lowest income (source: ABS—https://www.abs.gov.au/ausstats/abs@.nsf/Lookup/4363.0.55.001Appendix502011-13).

b Based on The Index of Relative Socio-economic Disadvantage (IRSD) Deciles 1–4, the index summarises the economic and social conditions of people within an area. Decile 1 indicates the most disadvantaged area with most households have low income, no qualifications and low skill occupations (source: ABS—https://www.abs.gov.au/ausstats/abs@.nsf/Lookup/by%20Subject/2033.0.55.001∼2016∼Main%20Features∼IRSD∼19).

c Based on the Kessler Psychological Distress Scale (K10) with scores 22—50 indicating high or very high psychological distress (source: ABS—https://www.abs.gov.au/ausstats/abs@.nsf/Lookup/by%20Subject/4363.0∼2014-15∼Main%20Features∼Kessler%20Psychological%20Distress%20Scale-10%20(K10)∼35).

The nine HLQ scale mean scores for both states were comparable, with Victoria slightly higher than NSW for all scales. For Scales 1 to 5 (score range of 1 to 4), all scales except Scale 5 Appraisal of health information had a score of > 3 for both states. For Scales 6 to 9 (score range of 1 to 5), all scale scores for both states were > 4. The results showed that people generally had good social and healthcare support, they were confident about accessing and understanding health information, but might have some issues around appraising health information. See HLQ scores of the two states in Table 1.

### Cluster analyses

The cluster analyses presented a different story. The results identified 11 clusters with varying health literacy patterns for NSW while 12 clusters were found to be the optimal representation of the Victorian sample. See Table 2 (NSW) and Table 3 (Victoria) for the clusters and related demographics. For NSW, the largest cluster was N4, representing 19.2% of the sample. People in this cluster were confident about their ability to engage with healthcare providers, navigate the healthcare system and understand health information but might not be as actively managing their health as some of the other clusters. This cluster had the second highest proportion (39%) of people who reported having low income compared to the other clusters. The smallest cluster was N9, making up 3.5% of the sample. People from this cluster had limited healthcare support and navigating the healthcare system could sometimes be challenging. Compared to the other clusters, this was the youngest cluster with a mean age of 47.3 years, and it had the highest proportion of people (36.1%) who reported experiencing anxiety.

**Table 2:** Health Literacy Profiles Based on the 11 Cluster Solutions of Respondents from New South Wales (NSW)

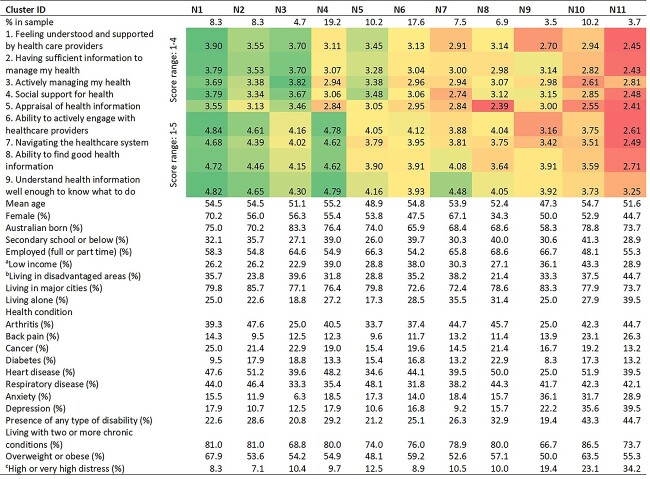

Note: The HLQ scale scores are colored using the traffic light system, with range of green indicating higher scores, range of yellow as medium scores and range of red as lower scores. Raw counts not presented due to low numbers for some of the cells with which percentages for these cells are presented as approximates to comply with Australian Bureau Statistics data release principles.

a Based on personal weekly income deciles 1—4, income deciles are ten populations groups classified from 1—10 in ascending order with the first decile represents the group with the lowest income (source: ABS—https://www.abs.gov.au/ausstats/abs@.nsf/Lookup/4363.0.55.001Appendix502011-13).

b Based on The Index of Relative Socio-economic Disadvantage (IRSD) Deciles 1—4, the index summarises the economic and social conditions of people within an area. Decile 1 indicates the most disadvantaged area with most households have low income, no qualifications and low skill occupations (source: ABS—https://www.abs.gov.au/ausstats/abs@.nsf/Lookup/by%20Subject/2033.0.55.001∼2016∼Main%20Features∼IRSD∼19).

c Based on the Kessler Psychological Distress Scale (K10) with scores 22—50 indicating high or very high psychological distress (source: ABS—https://www.abs.gov.au/ausstats/abs@.nsf/Lookup/by%20Subject/4363.0∼2014-15∼Main%20Features∼Kessler%20Psychological%20Distress%20Scale-10%20(K10)∼35).

**Table 3:** Health Literacy Profiles Based on the 12-Cluster Solution of Respondents from Victoria

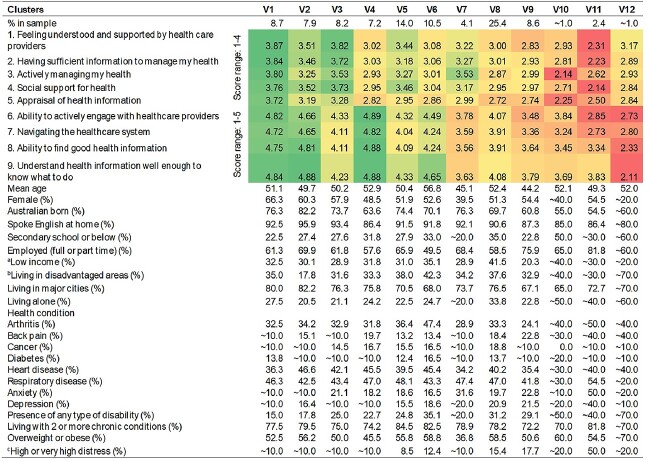

Note: The HLQ scale scores are colored using the traffic light system, with range of green indicating higher scores, range of yellow as medium scores and range of red as lower scores. Raw counts not presented due to low numbers for some of the cells with which percentages for these cells are presented as approximates to comply with Australian Bureau Statistics data release principles.

a Based on personal weekly income deciles 1–4, income deciles are ten populations groups classified from 1 to 10 in ascending order with the first decile represents the group with the lowest income (source: ABS—https://www.abs.gov.au/ausstats/abs@.nsf/Lookup/4363.0.55.001Appendix502011-13).

b Based on The Index of Relative Socio-economic Disadvantage (IRSD) Deciles 1–4, the index summarizes the economic and social conditions of people within an area. Decile 1 indicates the most disadvantaged area with most households have low income, no qualifications and low skill occupations (source: ABS—https://www.abs.gov.au/ausstats/abs@.nsf/Lookup/by%20Subject/2033.0.55.001∼2016∼Main%20Features∼IRSD∼19).

c Based on the Kessler Psychological Distress Scale (K10) with scores 22–50 indicating high or very high psychological distress (source: ABS—https://www.abs.gov.au/ausstats/abs@.nsf/Lookup/by%20Subject/4363.0∼2014-15∼Main%20Features∼Kessler%20Psychological%20Distress%20Scale-10%20(K10)∼35).

For Victoria, about a quarter of the sample (25.4%) belonged to cluster V8, the largest cluster. People from this cluster might not be thinking much about their health at the moment. This cluster had the highest proportion of people (41.5%) with low income among all clusters. The smallest cluster was V12, which represented ~1% of the sample. People from this cluster were facing challenges in getting health support and accessing or understanding health information but still attempted to manage their health. About 80% of the people in this cluster were male and ~70.0% of them were living with two or more chronic conditions and were overweight or obese.

While clusters N1, N2 and N3 from NSW had higher scale scores across the nine HLQ scales, the rest of the clusters presented various combinations of lower scores in certain scales, and they made up 78.7% of the NSW sample. Victoria also had similar results with clusters V1, V2 and V3 having higher scale scores across the nine scales and the remaining clusters having lower scores in some of the scales, and represented 75.2% of the Victorian sample. Comparing the health literacy patterns of the two states, six clusters from each state had similar patterns but five clusters from NSW and six clusters from Victoria were unique to the respective states. The six clusters with comparable patterns tended to have higher scores than those that were unique to each state. See Table 4 for descriptions of the clusters with cluster descriptions in bold representing clusters with similar patterns.

**Table 4:** Health Literacy Patterns of Respondents from New South Wales (NSW) and Victoria

Cluster ID	% in sample	Health literacy pattern
New South Wales		
N1	8.3%	**Confident in using health information and services with good support**
N2	8.3%	**Willing to leave healthcare and thinking about health in professional hands**
N3	4.7%	**Good health and social support but not proactively seeking healthcare**
N4	19.2%	**Not doing much now but confident that they can when they need to**
N5	10.2%	**Have good health and social support but not thinking much about health**
N6	17.6%	**Not thinking about health much at the moment**
N7	7.5%	Inadequate social and healthcare support and sometimes not confident in finding good information
N8	6.9%	Health literacy style based on interacting with others rather than searching for and understanding information for themselves, but the interactions may be breaking down
N9	3.5%	Limited healthcare support and have difficulty navigating the healthcare system but generally understand health information well
N10	10.2%	Inadequate social and healthcare support, having difficulty in finding and understanding health information and not managing health actively
N11	3.7%	Low trust and confidence in self and others
Victoria		
V1	8.7%	**Confident in using health information and services with good support**
V2	7.9%	**Willing to leave healthcare and thinking about health in professional hands**
V3	8.2%	**Good health and social support but not proactively seeking healthcare**
V4	7.2%	**Not doing much now but confident that they can when they need to**
V5	14.0%	**Have good health and social support but not thinking much about health**
V6	10.5%	Adequate health and social support but sometimes find health information confusing
V7	4.1%	Actively managing health but interactions with healthcare systems maybe breaking down
V8	25.4%	**Not thinking much about health at the moment**
V9	8.6%	Inadequate access to suitable health information and services
V10	2.2%	Not thinking about health with inadequate access to appropriate health information and services
V11	2.4%	Lack of social support and difficulty engaging with healthcare system
V12	1.0%	Attempting to manage health independently with limited healthcare support

Note: Health literacy patterns in bold are clusters with similar patterns for both New South Wales and Victoria.

## Discussion

### Main findings of this study

Using the 2018 Australian national health literacy survey data, this study applied cluster analysis to determine if this statistical method can provide more in-depth insights into people’s health literacy than descriptive statistics. The full sample HLQ scale mean scores of the two states were found to be comparable at the overall descriptive level, but the cluster analyses revealed 11 clusters in NSW and 12 clusters in Victoria, with five clusters and six clusters unique to NSW and Victoria, respectively. Importantly, the results uncovered that about two-third of respondents in each state were experiencing certain challenges in one or more health literacy dimensions.

What is already known about this topic?

Reporting average scores of survey tools is usual practice in population health research. In a systematic review of health literacy tools, Haun *et al.*  ^14^ identified 51 instruments, 47 of which presented the results as a sum score or percentage of correct responses or a statistical score or a weighted score or time of the full sample. Also, 14 of these tools further classified respondents into arbitrary categories such as high/adequate, marginal, inadequate/limited/low health literacy. Only four tools used scale scores, one of them was the HLQ. By using the HLQ, the ABS found that Australians generally felt ‘positive’ about their health literacy based on the interpretation drawn from the full sample nine scale mean scores. This is a logical conclusion of a population health literacy survey because most people are not experiencing daily health or health literacy crisis, and are not presenting to hospitals for emergency diagnosis or information. In contrast, many health literacy surveys apply arbitrary cutoffs to make claims that over half of populations have inadequate health literacy,^37–39^ which is incongruent with public health, but generates scientifically fraught conclusions and blaming of individuals, yet the procedure enables dramatic and often widely publicized claims by the respective survey developers and users.^5^

What this study adds?

Measuring health literacy is an important step to inform health literacy actions and strategies to address health inequities. Based on the findings from the ABS survey using the approach of population average scores, an assumption may be that the overall population may potentially benefit only from improvements in their evaluation skills of health information. However, population groups are never homogenous and the cluster analyses across two different states revealed two very different patterns of health literacy. While three clusters from each state demonstrated strengths across the nine health literacy dimensions, representing 21.3% and 24.8% of the NSW and Victoria sample, respectively, >75% of the samples from both states are facing one or more challenges in different health literacy dimensions. If only interventions related to increasing evaluation skills of health information as indicated by the average scores are implemented, the many other health literacy challenges of >75% of the two groups will be overlooked, potentially leading to the widening of health disparities instead of promoting health equity.

While the mean scores of the two states are comparable, Victoria has slightly higher mean scores across all nine scales than NSW. This may give the impression that Victorians may be in a slightly better position in terms of health literacy over their counterparts in NSW. However, the cluster analyses revealed a different story. When comparing all the 11 and 12 clusters from both states, the Victorian cluster V11 had the lowest scores for Scale 1 Feeling understood and supported by healthcare providers, Scale 2 Having sufficient information to manage my health, and Scale 4 Social support for health. Cluster V10 had the lowest scores for Scale 3 Actively managing my health and Scale 5 Appraisal of health information, and Cluster V9 had the lowest scores for Scale 8 Ability to find good health information and Scale 9 Understand health information well enough to know what to do. NSW only had one cluster, N11, which had the lowest scores for Scale 6 Ability to actively engage with healthcare providers and Scale 7 Navigating the healthcare system.

Based on the cluster analyses, health literacy actions can be tailored to the specific needs of subgroups within a population such that these groups will not be left behind by the usual one-size-fits-all type of interventions. Following the Ophelia process protocol,^20^ these health literacy profiles are usually used to generate vignettes that are presented at co-design workshops with community members and frontline health professionals to generate ideas for building fit-for-purpose actions that are relevant to the local context.^20^ The NSW cluster analysis results, combined with further data about mental health, were used in the ‘Developing Mental Health Literacy Responsiveness Education and Training’ project by the Mental Health Commission of New South Wales.^40^ The results led to the generation of vignettes, with both community members and health professionals agreeing that they were a good representation of the people in their community. Health literacy action areas to improve the mental health literacy responsiveness of organizations and services in NSW were developed and implemented based on these data-driven vignettes.^40^

### Limitations of the study

Currently, there is no consensus on how to best conduct a cluster analysis. The method we used allows for exploration of optimal cluster solutions instead of specifying a ‘pre-determined’ number of clusters, as in nonhierarchical cluster analysis, which is usually unknown to the researchers before the analysis. Another common critique of cluster analysis is the subjectivity involved in deciding on the optimal cluster solution and a different approach, the latent class clustering methods, has been suggested. The approach uses a model-based method to determine the optimal number of clusters using model fit criteria.^41^ However, some subjectivity in the analytical choices are still inevitable.^42^ Future research can explore a range of clustering approaches to ensure that clinically and socially meaningful population subgroups are identified, including subgroups with complex and perhaps infrequent health literacy challenges. Future research will help to uncover what public health research and practice methods leaves no one behind.

This study was not able to identify or hypothesize the causes of the differences between the clusters or the states. Studies using much larger samples sizes, longitudinal data, and linkage to hospitalizations, use of other health services and pharmaceutical use patterns will need to be undertaken. Such studies will enable an understanding of the relative risk of different pattern and potentially evidence-based thresholds to better inform policy and practice.

## Conclusions

By moving beyond the averages, this study identified 11 and 12 subgroups of Australians across NSW and Victoria, respectively, with most clusters possibly at higher risk of poor health outcomes and being left behind. The cluster analysis provides a more nuanced understanding of the range of health literacy patterns within a population and paves the way to inform tailored actions for health literacy development. The results demonstrate that cluster analysis can be used in public health to capture the diversity of people’s health literacy and promote health equity.^5^ This method is also expected to be beneficial for health literacy studies of smaller sample size or studies in resource-poor settings where health professionals may have limited statistical skills. To leave no one behind, public health and health literacy research must identify the different strengths and challenges of subgroups and make sure the voices of people are heard, so that tailored health actions can be developed to generate health equity.

## Supplementary Material

JPH-23-1499_R2_Supplementary_Data_1_R2_fdae165

Cheng_et_al_Cluster_Analyais_for_Health_Equity_Supplementary_Data_2_R1_fdae165

## Data Availability

The data are available in the Australian Bureau of Statistics DataLab. It will be available upon request following training through the Australian Bureau of Statistics.

## Notes

**Christina Cheng,** Dr

**Shandell Elmer,** A/Prof

**Roy Batterham,** A/Prof

**Melanie Hawkins,** Dr

**Richard H Osborne,** Prof
