# Supplementary material for: Measuring health literacy to inform actions to address health inequities: a cluster analysis approach based on the Australian national health literacy survey
Source: J Public Health (Oxf). 2024 Aug 4;46(4):e663–74. doi: 10.1093/pubmed/fdae165 (PMC11637599; doi:10.1093/pubmed/fdae165)
Supplement: JPH-23-1499_R2_Supplementary_Data_1_R2_fdae165 [file jph-23-1499_r2_supplementary_data_1_r2_fdae165.docx]

# Supplementary Data S1

**Manuscript Title:** Measuring health literacy to inform actions to address health inequities: a cluster analysis approach based on the Australian national Health Literacy Survey

# Table S1. Health Literacy Questionnaire (HLQ) scale descriptions

| **HLQ scale** | **Lower level of construct** | **Higher level of construct** |
| --- | --- | --- |
| **1. Feeling understood and supported by healthcare providers** | Unable to engage with doctors and other healthcare providers. Doesn’t have a regular healthcare provider and/or has difficulty trusting healthcare providers as a source of information and/or advice. | Has an established relationship with at least one healthcare provider who knows them well and who they trust to provide useful advice and information and to assist them to understand information and make decisions about their health. |
| **2. Having sufficient information to manage my health** | Feels that there are many gaps in their knowledge and that they don't have the information they need to live with and manage their health concerns. | Feels confident that they have all the information that they need to live with and manage their condition and to make decisions. |
| **3. Actively managing my health** | Doesn’t see their health as their responsibility, they are not engaged in their healthcare and regard healthcare as something that is done to them. | Recognises the importance and are able to take responsibility for their own health. They proactively engage in their own care and make their own decisions about their health. They make health a priority. |
| **4. Social support for health** | Completely alone and unsupported for health. | A person’s social system provides them with all the support they want or need for health. |
| **5. Appraisal of health information** | No matter how hard they try, they cannot understand most health information and get confused when there is conflicting information. | Able to identify good information and reliable sources of information. They can resolve conflicting information by themselves or with help from others. |
| **6. Ability to actively engage with healthcare providers** | Are passive in their approach to healthcare, inactive i.e., they do not proactively seek or clarify information and advice and/or service options. They accept information without question. Unable to ask questions to get information or to clarify what they do not understand. They accept what is offered without seeking to ensure that it meets their needs. Feel unable to share concerns. Does not have a sense of agency in interactions with providers. | Is proactive about their health and feels in control in relationships with healthcare providers. Is able to seek advice from additional healthcare providers when necessary. They keep going until they get what they want. Empowered. |
| **7. Navigating the healthcare system** | Unable to advocate on their own behalf and unable to find someone who can help them use the healthcare system to address their health needs. Does not look beyond obvious resources and has a limited understanding of what is available and what they are entitled to. | Able to find out about services and supports so they get all their needs met. Able to advocate on their own behalf at the system and service level. |
| **8. Ability to find good health information** | Cannot access health information when required. Is dependent on others to offer information. | Is an 'information explorer'. Actively uses a diverse range of sources to find information and is up to date. |
| **9. Understanding health information well enough to know what to do** | Has problems understanding any written health information or instructions about treatments or medications. Unable to read or write well enough to complete medical forms. | Is able to understand all written information (including numerical information) in relation to their health and able to write appropriately on forms where required. |
