# Supplementary material for: Measuring health literacy to inform actions to address health inequities: a cluster analysis approach based on the Australian national health literacy survey
Source: J Public Health (Oxf). 2024 Aug 4;46(4):e663–74. doi: 10.1093/pubmed/fdae165 (PMC11637599; doi:10.1093/pubmed/fdae165)
Supplement: Cheng_et_al_Cluster_Analyais_for_Health_Equity_Supplementary_Data_2_R1_fdae165 [file cheng_et_al_cluster_analyais_for_health_equity_supplementary_data_2_r1_fdae165.docx]

# Supplementary Data S2

**Manuscript Title:** Measuring health literacy to inform actions to address health inequities: a cluster analysis approach based on the Australian national Health Literacy Survey

# Table S2. Characteristics of a ‘typical’ New South Wales and a ‘typical’ Victorian

| **Characteristics*** | **The ‘Typical’ New South Wales** | **The ‘Typical’ Victorian** |
| --- | --- | --- |
| Age | 38 | 37 |
| Sex | Female | Female |
| Country of birth of person | Australia | Australia |
| Country of birth of parents | At least one parent born overseas | At least one parent born overseas |
| Language spoken at home | English | English |
| Ancestry 1^st^ response | English | English |
| Social marital status | Married in a registered marriage | Married in a registered marriage |
| Family composition | Couple family with children | Couple family with children |
| Count of all children in family | Two children in family | Two children in family |
| Highest year of school completed | Year 12 or equivalent | Year 12 or equivalent |
| Unpaid domestic work: number of hours | 5 to 14 hours | 5 to 14 hours |
| Number of motor vehicles | Two vehicles | Two vehicles |
| Number of bedrooms in private dwelling | Three bedrooms | Three bedrooms |
| Tenure type (dwelling count) | Owned outright | Owned with a mortgage |

Source: <https://www.abs.gov.au/websitedbs/D3310114.nsf/home/2016+Census+National>

*All characteristics are derived from the 2016 ABS Census data and based on the mode, the most commonly occurring value in a distribution, except for age which is based on the median.
